# Causal effect heterogeneity estimation using summary statistics

**DOI:** 10.21203/rs.3.rs-8589460/v1

**Published:** 2026-01-14

**Authors:** Yadong Yang, Minxi Bai, Jiacheng Miao, Stephen Dorn, Jonathan Haugstad, Jin Liu, Qiongshi Lu, Xingjie Shi

**Affiliations:** 1 KLATASDS-MOE, Academy of Statistics and Interdisciplinary Sciences, School of Statistics, East China Normal University; 2 Department of Biostatistics & Medical Informatics, University of Wisconsin–Madison; 3 School of Data Science, The Chinese University of Hong Kong, Shenzhen

## Abstract

Mendelian randomization (MR) has swiftly gained popularity as a tool for causal inference in genetic epidemiology. However, existing MR methods focus exclusively on estimating the average causal effect and cannot quantify its heterogeneity, posing a major methodological limitation and impeding context-dependent causal findings. Here, we introduce MEndelian Randomization for Linear INteraction (MERLIN), a unified Bayesian framework that jointly estimates the average and context-dependent causal effects using summary data from genome-wide association and interaction studies. Through extensive simulation analyses, we demonstrate the improved power, robustness, and broad utility of MERLIN versus existing methods. We show MERLIN was able to identify sex-specific causal effects of schizophrenia on brain imaging traits, a male-specific causal effect of testosterone on bipolar disorder, and age-dependent causal effects of metabolic biomarkers on coronary artery disease risk. These results illustrate the transformative potential of summary-data-based inference for causal heterogeneity. Together, MERLIN provides a powerful and practical framework for investigating causal effect heterogeneity using summary-level observational data and greatly enhances our capability to elucidate complex disease etiology.

## Introduction

Dissecting the causal underpinnings of complex human traits remains a fundamental goal in genetic epidemiology. Mendelian randomization (MR) has emerged as a powerful tool for causal inference. Through its leveraging of genetic variants, typically single-nucleotide polymorphisms (SNPs), as instrumental variables (IVs), MR can uncover the potential causal effect of exposure on an outcome while mitigating the confounding inherent in observational data^1-4^. Importantly, MR requires only summary statistics from genome-wide association studies (GWAS)—a feature that has contributed to its adoption. Over the past 20 years, GWAS have provided a vast amount of publicly available summary statistics data, fueling the widespread application of two-sample MR^5-9^.

However, MR typically estimates an average causal effect and implicitly assumes this effect is homogeneous in the population^4^. This assumption is often violated in practice, as biological processes exhibit context dependency. Thus the causal impact of an exposure may differ substantially across subpopulations along with modifiers such as environmental conditions, demographics, or distinct genetic backgrounds (often termed effect modification or heterogeneity)^10,11^. For instance, the causal effect of body mass index (BMI) on testosterone levels has been shown to vary markedly between the sexes, manifesting as a negative association in males but a positive association in females^12^. Identifying such heterogeneity in causal inference is crucial for understanding biological mechanisms and advancing precision medicine, as the efficacy of interventions may be restricted to certain population strata^13^. Conversely, ignoring effect heterogeneity can result in erroneous conclusions on the presence, absence, or magnitude of a causal relationship. As we demonstrate in this paper, failure to account for causal effect heterogeneity biases estimates of the average effect and obscures context-dependent effects in MR analyses^14-18^.

Current MR methods cannot simultaneously evaluate the average effects and context-dependent effects of an exposure, especially when applied to summary-level data. Although effect heterogeneity can, in principle, be investigated via sample stratification—an estimation of the causal effects within strata defined by environmental factors and subsequent tests for differences across strata^11,19-21^—this approach presents several methodological challenges. When environmental modifiers are continuous, defining appropriate strata becomes challenging and arbitrary^22^. Stratification also reduces the statistical power, as sample division into subgroups leads to smaller effective sample sizes^23^. Several methods have been introduced to model effect heterogeneity, but they require individual-level data^22,24^ often unavailable in genomic studies due to privacy and policy constraints^25^. Another critical, frequently overlooked challenge occurs when a trait’s additive genetic effects and context-dependent genetic effects are correlated, which can substantially bias standard MR estimates. We will further illustrate these points in later sections.

To address these challenges, we introduce MERLIN (MEndelian Randomization for Linear INteraction), a statistical framework enabling the unified estimation of the average causal effect and its heterogeneity across either discrete or continuous modifier contexts using only summary-level data. MERLIN uniquely leverages summary statistics from GWAS (for average genetic effects on exposure/outcome) and genome-wide interaction studies (GWIS; for SNP-by-context interaction effects) as inputs within a coherent joint model. This approach obviates the need for sample stratification and access to individual-level data. It provides robust estimates even under the presence of correlations between GWAS and GWIS inputs and arbitrary sample overlaps between exposure and outcome studies—complex but realistic data scenarios that can lead to bias in other methods. Furthermore, by leveraging a broader set of instruments informed by either additive or interaction effects, MERLIN provides increased statistical power, even under conventional MR settings. We demonstrate the performance of our method through extensive simulations and real-data applications.

## Results

### The MERLIN framework for causal effect estimation

**Figure 1.**
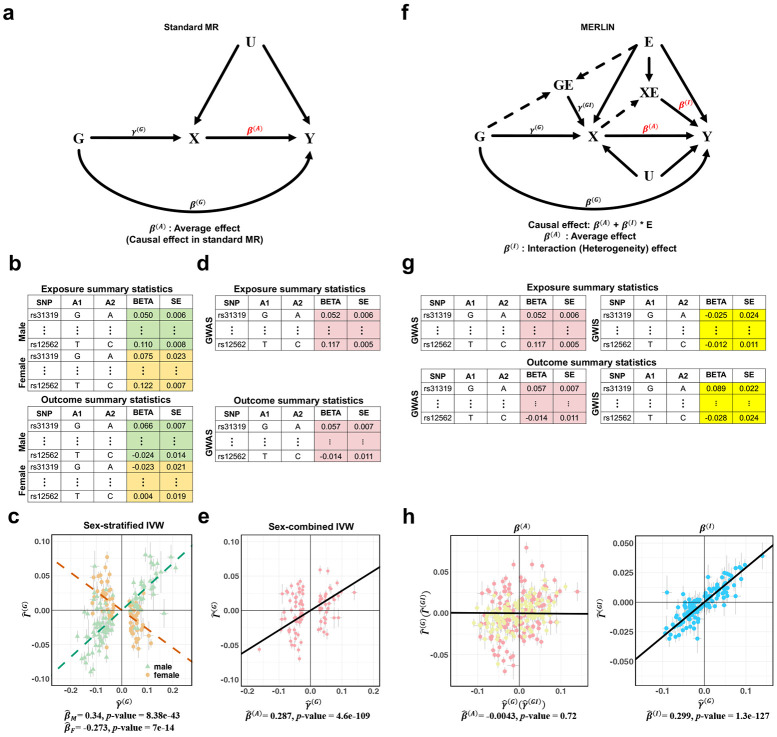
Conceptual frameworks of MR and MERLIN. We compare these methods using a simulated example with correlated GWAS and GWIS effects for the exposure. **(a-e)** MR workflow and its limitations under causal effect heterogeneity. **(a):** causal diagram for standard MR, where genetic instruments (G) for exposure (X) are used to estimate its causal effect ($\beta^{\left(A\right)}$) on an outcome (Y), accounting for unmeasured confounders (U) and potential pleiotropy ($\beta^{\left(G\right)}$). Some MR methods rely on the assumption of no horizontal pleiotropy. **(b):** Sex-stratified MR requires GWAS summary statistics with sex-stratification. **(c):** Sex-stratified IVW reveals the distinct effects in males (${\hat{\beta }}_{M}=0.340$, green datapoints in plot) and females (${\hat{\beta }}_{F}=-0.273$, orange datapoints). **(d):** MR typically requires only GWAS summary statistics without stratification. **(e):** An illustrative simulation example in which the true sex-specific effects are inverted ($\beta_{M}=0.3$, $\beta_{F}=-0.3$; true average main effect $\beta^{\left(A\right)}=0$). Standard IVW applied to sex-combined data (pink datapoints) yields a biased average effect estimate (${\hat{\beta }}^{\left(A\right)}=0.287$, $P=4.6\times 10^{-109}$, the black line). **(f-h)** The MERLIN framework. **(f):** Schematic representation of the MERLIN model. MERLIN jointly models the average causal effect ($\beta^{\left(A\right)}$) and the causal interaction effect ($\beta^{\left(I\right)}$). **(g):** Required input summary statistics for MERLIN. These include GWAS and GWIS results (i.e., point estimates and standard errors) for exposure and outcome traits. **(h):** Illustrative MERLIN output for the same data scenario as in (a), demonstrating its ability to disentangle average and interaction effects. MERLIN accurately estimates both the null average effect (${\hat{\beta }}^{\left(A\right)}=-0.0043$, P=0.72) and the true interaction effect (${\hat{\beta }}^{\left(I\right)}=0.299$, $P=1.3\times 10^{-127}$). In the scatter plots, each datapoint corresponds to an individual SNP instrument. Left plot: Displays outcome GWAS effects (${\hat{\Gamma }}^{\left(G\right)}$) and GWIS effects (${\hat{\Gamma }}^{\left(\mathrm{GI}\right)}$) versus exposure GWAS effects (${\hat{\gamma }}^{\left(G\right)}$) and GWIS effects (${\hat{\gamma }}^{\left(\mathrm{GI}\right)}$), and datapoint colors distinguish GWAS (pink) and GWIS (yellow); the slope of the regression line corresponds to the average effect estimate, ${\hat{\beta }}^{\left(A\right)}$. Right plot: Displays outcome GWIS effects (${\hat{\Gamma }}^{\left(\mathrm{GI}\right)}$) versus exposure GWAS effects (${\hat{\gamma }}^{\left(G\right)}$) (blue); the slope of the regression line corresponds to the interaction effect estimate, ${\hat{\beta }}^{\left(I\right)}$.

Standard MR estimates the average causal effects (Fig. 1a), typically using GWAS summary statistics for both exposure (${\hat{\gamma }}^{\left(G\right)}$) and outcome (${\hat{\Gamma }}^{\left(G\right)}$) traits as input. However, its performance under the presence of causal effect heterogeneity can be suboptimal. For example, in a simple scenario where the causal effects of exposure X on an outcome Y have the same magnitude but are inverted in males and females (i.e., $\beta_{M}=-\beta_{F}$), MR researchers commonly have two intuitions: (1) MR cannot identify the causal effect difference between sexes when applied to sex-combined GWAS data, but it will at least correctly estimate the null average causal effect $\beta^{\left(A\right)}=0$, and (2) MR can estimate sex-specific causal effects (i.e., $\beta_{M}$ and $\beta_{F}$) if applied to sex-stratified GWAS (Fig. 1b). Later, we will show that, while the second intuition is true for binary modifiers (e.g., biological sex, results shown in Fig. 1c), it produces less efficient estimates compared to a unified approach like MERLIN and cannot handle continuous modifiers. We also show that, perhaps surprisingly, even the first intuition is not always correct. If additive genetic effects (i.e., GWAS associations) and SNP-by-sex interaction effects (i.e., GWIS signals) on the exposure are correlated, a common phenomenon with clear interpretations (see recent work on polygenic gene-environment interaction inference^26^), MR can become substantially biased. We illustrate this in Fig. 1d-e (more extensive results are provided in later sections). In this simulation, the causal effects are opposite in males and females (e.g., $\beta_{M}=0.3$ and $\beta_{F}=-0.3$), yielding an average effect of $\beta^{\left(A\right)}=0$ in our framework. However, when applied to sex-combined summary data (Fig. 1d), MR yields a biased average effect estimate that is significantly different from zero (${\hat{\beta }}^{\left(A\right)}=0.287$, $P=4.6\times 10^{-109}$, shown in Fig. 1e). This highlights the need for a unified approach that can robustly estimate both the average and context-dependent effects.

We designed MERLIN to address these challenges (Fig. 1f). MERLIN extends the MR model by incorporating the causal effect heterogeneity explained by the modifier E. Briefly, for an individual i, we have:

$$\;X_{i}=\sum_{j=1}^{M}G_{ij}\gamma_{j}^{\left(G\right)}+E_{i}\gamma^{\left(E\right)}+\sum_{j=1}^{M}G_{ij}E_{i}\gamma_{j}^{\left(GI\right)}+U_{i}\eta_{X}+\epsilon_{X,i},\;Y_{i}=X_{i}\beta^{\left(A\right)}+\sum_{j=1}^{M}G_{ij}\beta_{j}^{\left(G\right)}+E_{i}\beta^{\left(E\right)}+X_{i}E_{i}\beta^{\left(I\right)}+U_{i}\eta_{Y}+\epsilon_{Y,i},$$

where $X_{i}$ and $Y_{i}$ are the covariate-adjusted exposure and outcome traits, $G_{ij}$ denotes the centered genotype of the j-th SNP, $E_{i}$ is the centered modifier variable, $U_{i}$ denotes the unmeasured confounders, and $\epsilon_{X}$ and $\epsilon_{Y}$ are random noise terms. The MERLIN framework introduces two key innovations. First, in addition to the average causal effect $\beta^{\left(A\right)}$, it allows the causal effect to be modified by E, denoted by the path (X×E→Y) with effect size $\beta^{\left(I\right)}$. We refer to this parameter as “the causal interaction” throughout this paper. Second, it allows SNPs to influence X via a gene-environment interaction (G×E→X); i.e., the genetic effects on exposure can be context-dependent, which also means that GWIS signals can be used as instruments in MERLIN.

Note that this model allows for standard horizontal pleiotropy (G→Y, effect $\beta^{\left(G\right)}$) and can be similarly extended to incorporate direct G×E effects on the outcome (G×E→Y). Details of this model and its more generalized forms are presented in Methods and **Supplementary Notes**. The average causal effect $\beta^{\left(A\right)}$ and causal interaction $\beta^{\left(I\right)}$ are the main parameters of interest in this paper.

Importantly, MERLIN only uses summary-level data as input (Fig. 1g). Specifically, it requires estimates of the main genetic effects on exposure (${\hat{\gamma }}^{\left(G\right)}$) and outcome (${\hat{\Gamma }}^{\left(G\right)}$) from GWAS and estimates of the G×E interaction effect on exposure (${\hat{\gamma }}^{\mathrm{GI}}$) and outcome (${\hat{\Gamma }}^{\left(\mathrm{GI}\right)}$) from GWIS. Under this formulation, the genetic effects on the outcome ($\Gamma^{\left(G\right)}$ and $\Gamma^{\left(\mathrm{GI}\right)}$) are functions of the genetic effects on exposure ($\gamma^{\left(G\right)}$ and $\gamma^{\left(\mathrm{GI}\right)}$), the causal parameters of interest ($\beta^{\left(A\right)}$ and $\beta^{\left(I\right)}$), and the horizontal pleiotropic effects ($\beta^{\left(G\right)}$), with the core relationships being defined by the following equations (Methods; **Supplementary Notes** for full model):

$$\;\Gamma^{\left(G\right)}=\beta^{\left(A\right)}\gamma^{\left(G\right)}+\beta^{\left(G\right)},\;\Gamma^{\left(GI\right)}=\beta^{\left(A\right)}\gamma^{\left(\mathrm{GI}\right)}+\beta^{\left(I\right)}\gamma^{\left(G\right)}.$$

In the **Supplementary Notes**, we also provide a strategy to estimate $\beta^{\left(A\right)}$ and $\beta^{\left(I\right)}$ without the outcome GWIS. This is of practical importance, as GWIS summary statistics data are not yet commonly available. Applied to the same illustrative scenario presented in Fig. 1c and Fig. 1e, MERLIN leverages the relationships between these four sets of summary statistics to disentangle the causal effects, providing estimates that are in line with the true parameters: a null average effect (${\hat{\beta }}^{\left(A\right)}=-0.0043$, P=0.72; true $\beta^{\left(A\right)}=0$) and a substantial interaction effect (${\hat{\beta }}^{\left(I\right)}=0.299$, $P=1.3\times 10^{-127}$; true $\beta^{\left(I\right)}=0.3$) (Fig. 1h). MERLIN thus mitigates the bias in the average effect estimation while directly quantifying effect modification ($\beta^{\left(I\right)}$). In the following sections, we demonstrate that this is a powerful and flexible framework that can handle both discrete and continuous modifiers and is robust to potential GWAS–GWIS effect correlations. We also present the effects transformation formula for binary phenotypes in the **Supplementary Notes**.

### Estimating average causal effect

We conducted simulation studies based on real genotype data from unrelated participants (N=337,056) in the UK Biobank (UKB) to evaluate MERLIN’s performance in estimating the average causal effect ($\beta^{\left(A\right)}$) and compared it with four MR methods: MR-LDP^5^, Robust Adjusted Profile Score (RAPS)^6^, Inverse Variance Weighted (IVW)^7^, and MR-Egger^8^. While IVW assumes no horizontal pleiotropy, MR-LDP, RAPS, and MR-Egger allow for some forms of it; however, all four methods assume the absence of causal interaction (i.e., $\beta^{\left(I\right)}=0$). Detailed simulation settings, including heritability values, induced correlations, phenotypic data generation from genotype data, and IV selection strategies for each method, are described in the Methods section.

**Figure 2.**
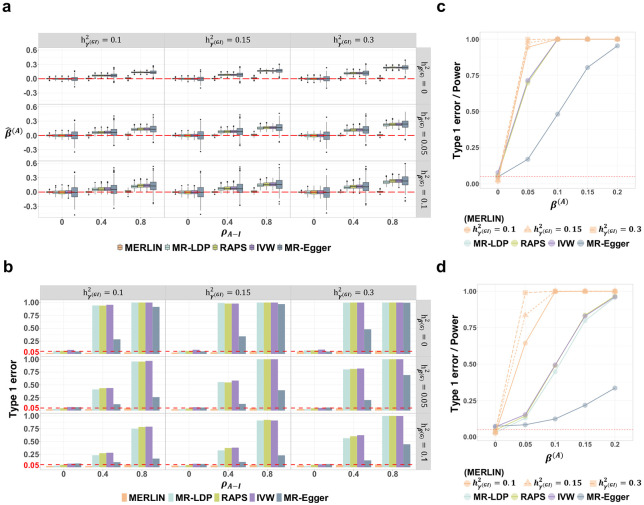
Simulation performance of MERLIN and standard MR methods for estimating the average causal effect ($\beta^{\left(A\right)}$). All simulations assumed a true interaction effect $\beta^{\left(I\right)}=0.3$ and were based on 500 replicates. MR methods included MR-LDP, RAPS, IVW, and MR-Egger. **(a)** Boxplots of average effect estimates ($\beta^{\left(A\right)}$) from all methods. Performance is shown across varying proportions of exposure variance explained by G×E effects ($h_{\gamma^{\left(\mathrm{GI}\right)}}^{2}=0.1,0.15,0.3$) and varying correlations between GWAS and GWIS effects ($\rho_{A-I}$; x-axis within each plot: 0, 0.4, 0.8). Scenarios are presented for true $\beta^{\left(A\right)}=0$ with horizontal pleiotropy ($h_{\beta^{\left(G\right)}}^{2}=0,0.05,0.1$). Dashed red lines indicate the true $\beta^{\left(A\right)}$ values. **(b)** Type I error rates for testing $\beta^{\left(A\right)}=0$, corresponding to the simulation conditions in panel (a). The dashed red line indicates the nominal 0.05 significance level. **(c, d)** Statistical power to detect $\beta^{\left(A\right)}\neq 0$ across several effect magnitudes, assuming $\rho_{A-I}=0$, and with $h_{\beta^{\left(G\right)}}^{2}=0$ and 0.1, respectively. The plot also shows type I error at $\beta^{\left(A\right)}=0$. Solid lines represent performance for all methods with $h_{\gamma^{\left(\mathrm{GI}\right)}}^{2}=0.1$. Dashed orange lines illustrate MERLIN performance with stronger G×E signals for exposure ($h_{\gamma^{\left(\mathrm{GI}\right)}}^{2}=0.15,0.3$).

First, we assessed the methods’ performance in the presence of causal interaction ($\beta^{\left(I\right)}=0.3$). When the additive genetic effects (GWAS signals) and SNPxE interaction effects (GWIS signals) were uncorrelated ($\rho_{A-I}=0$), all methods provided unbiased estimates of $\beta^{\left(A\right)}$ (Fig. 2a; **Supplementary Fig. S1**) and exhibited appropriately controlled type I error rates (Fig. 2b). However, introducing a correlation between the GWAS and GWIS effects ($\rho_{A-I}=0.4$ and 0.8) led to biased $\beta^{\left(A\right)}$ estimates and substantially inflated type I errors for MR-LDP, RAPS, IVW, and MR-Egger, with bias worsening as correlation increased (Fig. 2a, 2b; **Supplementary Fig. S1**). In contrast, MERLIN maintained unbiased estimations and well-calibrated type I error control across all correlation levels. The effect correlation between GWAS and GWIS has an interpretation of polygenic gene-environment interaction^26^. Our results demonstrate MERLIN’s robustness to such interactions.

Next, we compared their statistical power to detect a non-zero average effect $\left(\beta^{\left(A\right)}>0\right)$ in this scenario. In the absence of horizontal pleiotropy $\left(h_{\beta^{\left(G\right)}}^{2}=0\right)$, MERLIN demonstrated greater power than the other methods. This advantage grew as the exposure’s GWIS signal strength $\left(h_{\gamma^{\left(GI\right)}}^{2}\right)$ increased from 0.1 to 0.3 (Fig. 2c). The superiority of MERLIN was even more evident in the presence of horizontal pleiotropy $\left(h_{\beta^{\left(G\right)}}^{2}=0.1\right)$. Under this condition, the power of IVW, RAPS, and MR-Egger decreased substantially, while that of MERLIN remained robust, resulting in a drastic improvement in performance (Fig. 2d).

We also compared these methods without causal interaction ($\beta^{\left(I\right)}=0$, **Supplementary Fig. S2** and **S3**). Under this scenario, all methods, including MERLIN, provided unbiased estimates of $\beta^{\left(A\right)}$ (**Supplementary Fig. S2a**, **S3**) and well-calibrated type I error rates (**Supplementary Fig. S2b**). We compared the methods’ statistical power under this condition, assuming no causal interaction. With uncorrelated GWAS/GWIS effects $\left(\rho_{A-I}=0\right)$ and in the absence of pleiotropy ($h_{\beta^{\left(G\right)}}^{2}=0$; **Supplementary Fig. S2c**), MERLIN demonstrated comparable power to MR-LDP, RAPS, and IVW. All methods were more powerful than MR-Egger. In the presence of horizontal pleiotropy $\left(h_{\beta^{\left(G\right)}}^{2}=0.1\right)$, MERLIN outperformed the other methods (**Supplementary Fig. S2d**). Notably, its power increased with stronger GWIS signals for exposure ($h_{\gamma^{\left(\mathrm{GI}\right)}}^{2}=0.15,0.3$), even when causal interaction $\beta^{\left(I\right)}$ was zero.

Together, these simulations demonstrate that MERLIN provides robust and unbiased estimates of the average causal effect $\beta^{\left(A\right)}$, maintaining accurate type I error control even under conditions that bias standard MR methods (e.g., under the presence of causal interaction $\beta^{\left(I\right)}\neq 0$, coupled with GWAS-GWIS correlation $\rho_{A-I}\neq 0$. Furthermore, MERLIN often achieves higher statistical power for detecting $\beta^{\left(A\right)}$. This power advantage is particularly evident in the presence of horizontal pleiotropy and when MERLIN can leverage informative GWIS signals, even if the true interaction effect $\beta^{\left(I\right)}$ is null.

### Causal interaction estimation under discrete and continuous modifiers

**Figure 3.**
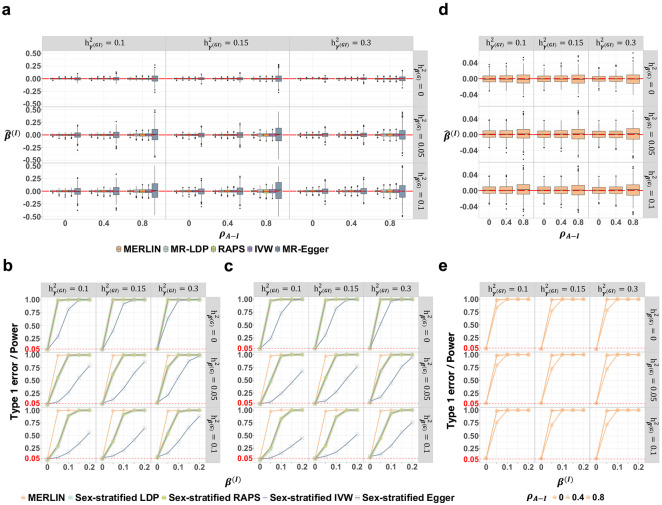
MERLIN accurately estimates causal interaction $\beta^{\left(I\right)}$ and demonstrates superior statistical power in simulations. All simulations assumed a true average effect $\beta^{\left(A\right)}=0$ and were based on 500 replicates. **(a)** Boxplots show the interaction effect estimates ($\beta^{\left(I\right)}$) when the true $\beta^{\left(I\right)}=0$ for a discrete modifier. Performance is shown across varying G×E signal strengths for exposure ($h_{\gamma^{\left(\mathrm{GI}\right)}}^{2}$); columns), correlations between GWAS and GWIS instrument effects ($\rho_{A-I}$; x-axis of boxplots), and levels of horizontal pleiotropy ($h_{\beta^{\left(G\right)}}^{2}$; rows). Dashed red lines indicate the true $\beta^{\left(I\right)}=0$. **(b)** Power comparison between all methods to detect $\beta^{\left(I\right)}\neq 0$ for a discrete modifier in scenarios with no GWAS–GWIS correlation ($\rho_{A-I}=0$) for varying horizontal pleiotropy ($h_{\beta^{\left(G\right)}}^{2}$) and G×E signal strengths ($h_{\gamma^{\left(\mathrm{GI}\right)}}^{2}$). **(c)** Power comparison as in (b) but in the presence of GWAS–GWIS correlation ($\rho_{A-I}=0.4$). **(d)** Boxplots show the MERLIN estimates for $\beta^{\left(I\right)}$ (true $\beta^{\left(I\right)}=0$) for a continuous modifier across conditions analogous to those in panel (a). **(e)** Statistical power of MERLIN to detect $\beta^{\left(I\right)}\neq 0$ for a continuous modifier. Power increases with true $\beta^{\left(I\right)}$ magnitude and G×E signal strength ($h_{\gamma^{\left(\mathrm{GI}\right)}}^{2}$), with type I error controlled, across various $\rho_{A-I}$ and $h_{\beta^{\left(G\right)}}^{2}$ values.

We next evaluated MERLIN’s performance when estimating the causal interaction effect ($\beta^{\left(I\right)}$), which quantifies the modification of effect by an environmental factor E. For simulations with a binary modifier variable (e.g., biological sex, coded as ±1), we compared MERLIN to sex-stratified MR approaches (i.e., estimating $\beta^{\left(I\right)}$ indirectly from sex-specific effects, see Methods). All methods yielded unbiased estimates of $\beta^{\left(I\right)}$ across various scenarios, including when the true $\beta^{\left(I\right)}=0$ (Fig. 3a; **Supplementary Fig. S4a**) and when $\beta^{\left(I\right)}=0.3$ (**Supplementary Fig. S5**), irrespective of the correlation between GWAS and GWIS effects $\left(\rho_{A-I}\right)$. All approaches also demonstrated well-calibrated type I error rates for testing $\beta^{\left(I\right)}=0$, irrespective of the correlation $\rho_{A-I}$ and average causal effect $\beta^{\left(A\right)}$ values (Fig. 3b-c; **Supplementary Fig. S4b-c** and **S6a-b**). We compared the statistical power of these methods to detect a non-zero $\beta^{\left(I\right)}$ with both uncorrelated (Fig. 3b; $\rho_{A-I}=0$) and correlated (Fig. 3c; $\rho_{A-I}=0.4$) GWAS-GWIS effects. MERLIN consistently demonstrated higher statistical power than sex-stratified LDP, RAPS, IVW, and MR-Egger, especially in the presence of horizontal pleiotropy ($h_{\beta^{\left(G\right)}}^{2}>0$, middle and bottom rows of each panel; Fig. 3b-c; **Supplementary Fig. S4b-c**, **S6**).

We also simulated scenarios with an unbalanced sex ratio: 25% males and 75% females. This resulted in a skewed modifier E, whose third moment after standardization was nonzero (see Methods). MERLIN continued to perform well under these conditions, providing unbiased estimates, well-calibrated type I error rates, and high statistical power for both the causal interaction ($\beta^{\left(I\right)}$) and average effect ($\beta^{\left(A\right)}$) (**Supplementary Fig. S7**).

Crucially, MERLIN also showcased robust performance when the modifier E was continuous, a scenario that precludes the use of existing methods based on sample stratification. MERLIN provided unbiased estimates of the interaction effect $\beta^{\left(I\right)}$, whether the average effect $\beta^{\left(A\right)}$ was absent (Fig. 3d) or present (**Supplementary Fig. S4d**). The type I error for $\beta^{\left(I\right)}$ was appropriately controlled (evident at $\beta^{\left(I\right)}=0$ in Fig. 3e, **S4e**). Furthermore, the statistical power of MERLIN to detect a non-zero interaction effect increased monotonically with the magnitude of the true effect ($\beta^{\left(I\right)}$ from 0 to 0.2) across different levels of GWAS–GWIS correlation ($\rho_{A-I}$) (Fig. 3e; **Supplementary Fig. S4e**).

These simulation results demonstrate that MERLIN accurately estimates the causal interaction $\beta^{\left(I\right)}$ for both discrete and continuous modifiers using summary statistics. Although stratified analysis can yield unbiased $\beta^{\left(I\right)}$ estimates for discrete modifiers, MERLIN provides a unified, statistically powerful solution that uniquely enables the investigation of effect modification by continuous variables based on summary data.

### MERLIN provides robust results under sample overlap

A common challenge during two-sample MR is the potential for sample overlap between exposure and outcome datasets, which can bias causal effect estimates. We performed simulations to evaluate MERLIN’s robustness to this issue. The simulations assumed complete sample overlap between the GWAS and GWIS of each trait (a realistic scenario in practice) and partial sample overlap between the exposure and outcome cohorts (see Methods for details).

MERLIN provided unbiased estimates for both the average effect ($\beta^{\left(A\right)}$) and the interaction effect ($\beta^{\left(I\right)}$), maintaining sufficient type I error control in all tested conditions, including when continuous modifiers were used (**Supplementary Figs. S8-S9**). In contrast, standard MR methods (excluding MR-Egger, which had low power) resulted in inflated type I errors for $\beta^{\left(A\right)}$ due to the exposure–outcome sample overlap (**Supplementary Fig. S8**).

### Benchmarking the performance of MERLIN using negative and positive controls

**Figure 4.**
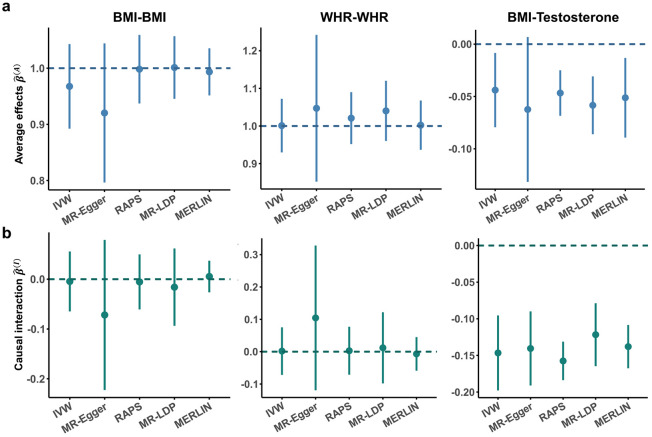
Performance of MERLIN and MR in negative and positive control analyses. Forest plots display comparisons of MERLIN with other MR methods (IVW, MR-Egger, RAPS, MR-LDP). **(a)** Average effect estimates (${\hat{\beta }}^{\left(A\right)}$) and **(b)** causal interaction estimates (${\hat{\beta }}^{\left(I\right)}$) for BMI on BMI, WHR on WHR, and BMI on testosterone. The left and middle columns represent negative control analyses (expected ${\hat{\beta }}^{\left(A\right)}\approx 1$, ${\hat{\beta }}^{\left(I\right)}\approx 0$; indicated by dashed lines). The right column shows the positive control analysis evaluating the effect of BMI on testosterone. In this analysis, MERLIN estimated the average effect as ${\hat{\beta }}^{\left(A\right)}=-0.0512$ (P=0.0083) and the causal interaction as ${\hat{\beta }}^{\left(I\right)}=-0.1379$ ($P=2.62\times 10^{-54}$), indicating significant moderation by sex. All data are presented as effect estimates with 95% CIs.

We evaluated the performance of MERLIN using real data incorporating negative and positive control scenarios^2,4^ defined by the expected causal interaction effect ($\beta^{\left(I\right)}$). The negative control analysis was designed to examine heterogeneity where none is expected (i.e., $\beta^{\left(I\right)}=0$). We used biological sex as the environment and examined the causal effects of BMI and waist-hip ratio (WHR) on themselves using summary statistics from different GWAS databases (i.e., UKB^19^ and GIANT consortium^27,28^) as the exposure and outcome inputs, respectively (**Supplementary Table S1** for data details). Assuming consistent scaling between exposure and outcome GWAS, we expected the true average causal effect to be one ($\beta^{\left(A\right)}=1$). All methods were able to identify a nonzero average effect of the trait on itself and produced 95% confidence intervals (CIs) that covered the true average effect of $\beta^{\left(A\right)}=1$ (Fig. 4a). With respect to the causal interaction estimation, both MERLIN and the sex-stratified MR methods produced estimates consistent with the null hypotheses ($\beta^{\left(I\right)}=0$) and yielded well-calibrated 95% CIs that encompassed zero. Compared to MR, MERLIN provided substantially narrower CIs (129% narrower in the BMI analysis, and 95% for WHR on average; Fig. 4; **Supplementary Table S2**), indicating its estimation of causal interaction was superior. In our sensitivity analysis of MERLIN, we applied 27 different instrument-selection settings. These were derived from a grid of three distinct thresholds for each parameter: GWAS P value, GWIS P value, and LD clumping (Methods). Similar findings were observed for different IV selection cutoffs (**Supplementary Fig. S10a**; **Supplementary Table S3-4**).

In our positive control analysis, we sought to replicate a previously reported sex difference in the causal effect of BMI on testosterone levels^12^. BMI summary statistics were obtained from the GIANT consortium,^29^ and testosterone level summary statistics were from UKB^30^ (**Supplementary Table S1** for data details). MERLIN identified a highly significant sex interaction in the causal effect (${\hat{\beta }}^{\left(I\right)}=-0.1379$, $P=2.62\times 10^{-54}$), consistent with expectations (Fig. 4b; **Supplementary Table S2**). MERLIN provided narrower CIs than standard MR methods (22% narrower on average; Fig. 4; **Supplementary Table S2**). We reconstructed sex-specific causal effects by transforming the MERLIN estimates of $\beta^{\left(A\right)}$ and $\beta^{\left(I\right)}$ (Methods) and obtained estimates showing inverted effect directions between sexes and an substantially elevated effect magnitude in males (${\hat{\beta }}_{M}=-0.1892$, $P=7.95\times 10^{-19}$; ${\hat{\beta }}_{F}=0.0867$, $P=4.89\times 10^{-5}$; **Supplementary Fig. S10b**), closely matching previous findings. The results were consistent across multiple IV selection thresholds (**Supplementary Fig. S10c**; **Supplementary Table S5**).

### Sex differences in causal effects between schizophrenia and brain imaging-derived phenotypes

Sex differences in schizophrenia (SCZ) prevalence, age of onset, and treatment response are well-documented, but the underlying neurobiological mechanisms are poorly understood. Brain image-derived phenotypes (IDPs) offer quantitative markers of brain structure and function that may reveal insights into sex-specific manifestations. To explore this, we applied MERLIN to systematically investigate the bidirectional causal relationships between SCZ and brain IDPs, testing for effect modification by sex.

We utilized large-scale GWAS summary statistics for SCZ (from Psychiatric Genomics Consortium)^31^ and brain IDPs (from the Oxford Brain Imaging Genetics Server)^32^ (**Supplementary Table S1**, **S6** for data details). After quality control and initial filtering^33^, we identified 49 IDPs showing significant genetic correlations with SCZ using LDSC (see Methods for details on data processing and GWIS generation; **Supplementary Table S7**; **Supplementary Fig. S11a**) and focused our bidirectional MR analyses on these pairs. We applied MERLIN and assessed the statistical significance using a Bonferroni-corrected threshold (Methods). The distributions of P values for bidirectional MR analysis using different methods is shown in **Supplementary Fig. S11b**.

**Figure 5.**
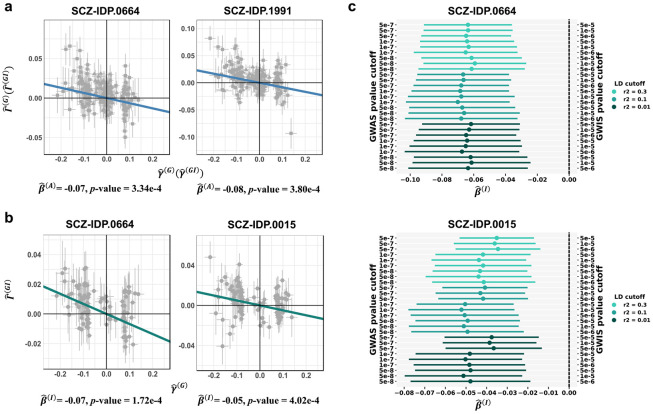
MERLIN identifies average and sex-specific causal effects between schizophrenia and brain imaging phenotypes. **(a)** MERLIN identified significant average effects in five phenotype pairs (only two representative pairs are shown here; results for all pairs are provided in **Supplementary Fig. S11c**). Scatter plots display outcome GWAS (${\hat{\Gamma }}^{\left(G\right)}$) and GWIS (${\hat{\Gamma }}^{\left(\mathrm{GI}\right)}$) effects versus exposure GWAS (${\hat{\gamma }}^{\left(G\right)}$) and GWIS (${\hat{\gamma }}^{\left(\mathrm{GI}\right)}$) effects; each point corresponds to an individual SNP instrument. The slope of the regression line corresponds to the average effect estimate, ${\hat{\beta }}^{\left(A\right)}$. **(b)** MERLIN identified significant causal interaction in two phenotype pairs. Scatter plots display outcome GWIS effects (${\hat{\Gamma }}^{\left(\mathrm{GI}\right)}$) versus exposure GWAS effects (${\hat{\gamma }}^{\left(G\right)}$); the slope of the regression line corresponds to the interaction effect estimate, ${\hat{\beta }}^{\left(I\right)}$. **(c)** Sensitivity analyses of MERLIN causal interaction estimates across different IV selection thresholds for SCZ-IDP.0664 and SCZ-IDP.0015. The estimates of $\beta^{\left(I\right)}$ remained consistent and systematically deviated from the null across varying GWAS *P* value thresholds (5 × 10^−8^, 1 × 10^−7^, 5 × 10^−7^), GWIS *P* value thresholds (5 × 10^−6^, 1 × 10^−5^, 5 × 10^−5^), and LD clumping r^2^ thresholds (0.1, 0.3, 0.5), demonstrating significant sex-related heterogeneity.

In the forward direction (i.e., IDPs’ effect on SCZ risk), MERLIN identified one significant causal association: increased volume of the left cerebellum cortex (IDP.0194) was associated with higher SCZ risk ($P=4.65\times 10^{-6}$; **Supplementary Fig. S11c**, **Supplementary Table S8**). After Bonferroni correction, no significant sex differences were detected for the effects of IDPs on SCZ. Conversely, in the reverse direction (i.e., SCZ risk’s effect on brain IDPs), MERLIN revealed significant average causal effects of SCZ liability on four different IDPs, including alterations in the parietal lobe and brainstem tracts (IDP.0664, IDP.1991, IDP.1992, IDP.1541; all $P<4\times 10^{-4}$; Fig. 5a, **Supplementary Fig. S11c**, **Supplementary Table S9**). Notably, MERLIN identified significant sex interactions in the causal impact of SCZ on two IDPs: the surface area of the left paracentral lobule (IDP.0664; interaction $P=1.72\times 10^{-4}$) and the volume of the left accumbens (IDP.0015; interaction $P=4.02\times 10^{-4}$) (Fig. 5b, **Supplementary Table S10**). These interaction findings remained robust across extensive sensitivity analyses employing 27 different instrument selection strategies (Methods, Fig. 5c, **Supplementary Table S11-12**). The comparative results from other MR methods and the sex-stratified analyses are provided in **Supplementary Fig. S12**.

Our MR findings causally link SCZ liability with alterations in specific cerebellar, sensorimotor, and corticofugal pathways. The identified causal role of the left cerebellar cortex (IDP.0194) in increasing SCZ risk is consistent with its involvement in cortico-cerebellar-thalamo-cortical circuits implicated in the disorder's cognitive symptoms^34^. Conversely, the causal effects of SCZ on the paracentral lobule (IDP.0664) and cerebral peduncles (IDP.1991, IDP.1992, IDP.1541) suggest that disease liability leads to alterations in sensorimotor and corticofugal pathways, aligning with evidence of white matter reorganization^35,36^ (see **Supplementary Notes** for a detailed discussion of these findings in the context of the existing literature). These findings imply that the neuropathological impact of SCZ on key sensorimotor and reward-system structures may be significantly greater in males, revealing potential causal pathways behind the known sex differences in the disorder^23^ and underscoring the importance of sex as a biological variable in SCZ research.

### Sex differences in causal effects of testosterone on neuropsychiatric disorders

Testosterone exhibits a strikingly low male–female genetic correlation ($r_{g}=0.12$)^21^, suggesting distinct genetic regulation mechanisms in men and women. Given that testosterone has been linked to a variety of psychiatric conditions^37,38^, we applied MERLIN to investigate the potential sex differences in these effects. We included six neuropsychiatric disorders in the analysis: bipolar disorder (BD), major depressive disorder (MDD), recurrent MDD (MDDR), obsessive-compulsive disorder (OCD), post-traumatic stress disorder (PTSD), and SCZ, leveraging large-scale GWAS data from UKB (for testosterone)^39^ and the Psychiatric Genomics Consortium^31,40,41^ (for neuropsychiatric disorders; see **Supplementary Table S1** for data summary).

**Figure 6.**
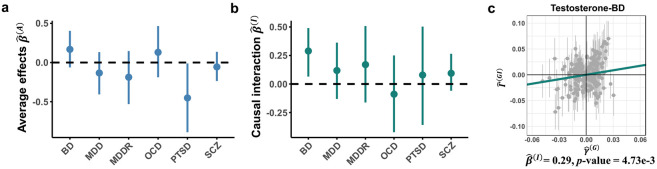
Causal effect of testosterone level on neuropsychiatric disorders. **(a-b)** MERLIN estimates for average causal effects (${\hat{\beta }}^{\left(A\right)}$) and sex interaction effects (${\hat{\beta }}^{\left(I\right)}$) of testosterone on six neuropsychiatric disorders, with 95% CIs. **(c)** MERLIN identified significant causal interaction in Testosterone-BD. The scatter plot displays outcome GWIS effects (${\hat{\Gamma }}^{\left(\mathrm{GI}\right)}$) versus exposure GWAS effects ($\gamma^{\left(G\right)}$); the slope of the regression line corresponds to the interaction effect estimate, ${\hat{\beta }}^{\left(I\right)}$.

Although MERLIN did not identify significant average causal effects after Bonferroni correction (Fig. 6a), we found a significant sex-specific causal effect of testosterone on BD (P=0.0047) (Fig. 6b-c; **Supplementary Table S13**). This effect was markedly stronger in males (${\hat{\beta }}_{M}=0.4564$, $P=5.4\times 10^{-3}$) than females (${\hat{\beta }}_{F}=-0.1224$, P=0.4557) (**Supplementary Fig. S13a**). The finding resonates with clinical observations of higher bipolar I disorder prevalence in men^42^ and its diagnostic patterns^43^. Potential biological mechanisms for this enhanced male risk could involve the sex-specific sensitivity caused by androgen receptor density or modulation by other sex hormones, such as estradiol, which influences the neurotransmission pathways implicated in BD^42,44,45^. The key finding of a male-specific testosterone effect on BD risk demonstrated robustness across extensive sensitivity analyses with varied instrument selection thresholds (Methods; **Supplementary Table S14**; **Supplementary Fig. S13b**), supporting the validity of the finding.

### Age-dependent causal effects of metabolic biomarkers on coronary artery disease

A key feature of the MERLIN framework is its capacity to analyze how continuous variables modify causal relationships. We applied MERLIN to examine the potential age-dependent causal effects of five established metabolic risk factors—BMI, diastolic and systolic blood pressure (DBP and SBP), low-density lipoprotein (LDL) cholesterol, and triglycerides (TG)—on the risk of developing coronary artery disease (CAD). These traits have been demonstrated to exert robust positive average effects on CAD in prior comprehensive MR analyses^46^. Understanding how the impact of these factors changes across the patient lifespan is critical for optimizing disease prevention strategies and early interventions. Our analyses utilized large-scale GWAS and gene-by-age (GxAge) interaction summary statistics from UKB and the All of Us Research Program^47^ (Methods; **Supplementary Table S1** for data details).

**Figure 7.**
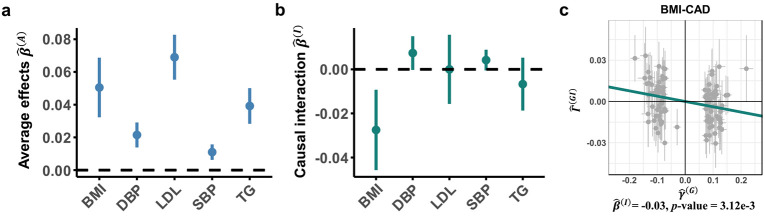
Age-dependent causal effects of metabolic biomarkers on coronary artery disease. **(a-b)** Forest plots display MERLIN estimates of average causal effects (${\hat{\beta }}^{\left(A\right)}$) and age-interaction effects (${\hat{\beta }}^{\left(I\right)}$) for five metabolic biomarkers (BMI, DBP, LDL, SBP, TG) on coronary artery disease (CAD) risk, with 95% CIs. Note: Y-axis scales for LDL and TG average effect estimates are adjusted for visualization. **(c)** MERLIN identified a significant causal interaction in BMI–CAD. The scatter plot displays outcome GWIS effects (${\hat{\Gamma }}^{\left(\mathrm{GI}\right)}$) versus exposure GWAS effects (${\hat{\gamma }}^{\left(G\right)}$); the slope of the regression line corresponds to the interaction effect estimate, ${\hat{\beta }}^{\left(I\right)}$.

After correction for multiple testing across the five biomarkers, MERLIN identified the average causal effects of BMI ($P=6.37\times 10^{-8}$), DBP ($P=4.23\times 10^{-8}$), LDL cholesterol ($P=1.21\times 10^{-21}$), SBP ($P=3.92\times 10^{-6}$), and TG ($P=1.80\times 10^{-12}$) on CAD risk. These results are consistent with their known roles^46-49^ as CAD risk factors (Fig. 7a; **Supplementary Table S15**). Our primary focus in this analysis was to investigate the presence of age-dependent effect heterogeneity. After Bonferroni correction, we identified that age significantly modified the causal impact of BMI ($P=3.12\times 10^{-3}$) on CAD risk (Fig. 7b-c). In contrast, we did not observe significant age-dependent heterogeneity for the other four metabolic traits (DBP, SBP, LDL, TG; all interaction P>0.05; **Supplementary Table S15**), suggesting that the attenuation of causal risk in later life may be a feature more specific to adiposity in this context. This highlights MERLIN’s capability to assess the modifications of effect due to continuous variables from summary statistics data, an analysis currently considered impossible with existing MR approaches.

These findings indicate that age-specific dynamics impact cardiovascular risk pathways. Specifically, the causal effect of elevated BMI on CAD risk was found to be attenuated with advancing age (${\hat{\beta }}^{\left(I\right)}=-0.0275$, $P=3.12\times 10^{-3}$). This provides a potential causal mechanism for the well-documented but controversial “obesity paradox”^52^, suggesting that the risk-conferring impact of higher BMI is most potent earlier in life. We noted that these findings were also robust to sensitivity checks (Methods; **Supplementary Table S16**, **Supplementary Fig. S13c**). To further validate the age-dependent effect of BMI on CAD risk, we performed a logistic regression analysis using individual-level data from the UKB. The model included BMI, age, and their interaction, and adjustments for potential confounders, including sex, education, diet, and physical activity (with age, sex, and BMI standardized). Consistent with our MR findings, the analysis demonstrated a significant positive association between BMI and CAD risk ($\hat{\beta }=0.2482$, $P<2\times 10^{-16}$), while the interaction term between BMI and age was statistically significant and negative ($\hat{\beta }=-0.0294$, $P<3\times 10^{-7}$) (**Supplementary Table S17**, **Supplementary Fig. S13d**). Notably, our results align with a recent large-scale study^53^, which revisited obesity thresholds in Chinese adults and similarly identified distinct age-specific patterns in cardiovascular risk. The convergence of our causal estimates in a European-ancestry cohort with their epidemiological findings in an Asian population underscores the robustness of this age-dependent effect. Collectively, these lines of evidence suggest that clinical guidelines may benefit from evolving towards age-stratified BMI targets to optimize cardiovascular prevention strategies.

## Discussion

In this study, we developed and evaluated MERLIN, a statistical framework that overcomes several critical limitations of MR by enabling the unified estimation of both the average causal effect ($\beta^{\left(A\right)}$) and the causal interaction ($\beta^{\left(I\right)}$). To the best of our knowledge, MERLIN is the first method to achieve this solely using summary-level data from GWAS and GWIS. In our extensive simulations, MERLIN provided accurate estimates and maintained appropriate type I error control for both parameters, often achieving greater statistical power than the comparator MR methods evaluated, particularly when leveraging informative GWIS signals in the presence of horizontal pleiotropy.

MERLIN provides several important methodological advances in the delineation of context-dependent causal mechanisms. First, it is a highly flexible framework that simultaneously considers the average effect of exposure on the outcome and the modification of this effect by other variables. To achieve this, it borrows the genetic IV idea from MR and expands on it to incorporate SNP-by-modifier interactions for both the exposure and outcome traits. MERLIN provides an accurate estimation of the average causal effect under conditions that often challenge existing MR approaches, such as when GWAS and GWIS effects are correlated (see recent work^26^ for a gene–environment interaction perspective on interpreting these correlations) or when there is sample overlap between the exposure and outcome cohorts. This framework also allows us to estimate the causal interaction effects of continuous modifiers, a key task that exceeds the abilities of existing methods. Second, the incredible progress made in complex trait genetics in the past decade have been partly driven by the expansion in GWAS consortia and the public release of GWAS summary statistics. The MR research field exemplifies this progress, with two-sample MR methodologies, requiring only GWAS summary statistics as input, forming the foundation of this success. However, when studying interactions and effect heterogeneity, current analytical strategies are largely based on (often small) individual-level datasets^54^. Miao et al. recently argued that those interested in gene–environment interactions should follow the playbook of complex trait genetics and start developing methods that only use summary statistics as input^26^. MERLIN advances this vision by introducing a solution to the estimation of causal effect heterogeneity using summary statistics alone (from GWAS and GWIS). The significance of this is two-fold: (1) the summary statistics-based approach ensures MERLIN’s broad applicability, especially given its robust performance in the face of technical issues such as sample overlap; (2) by creating tools that leverage GWIS summary-level data, more consortia can conduct GWIS meta-analysis and release their summary statistics, creating a virtuous circle that truly advances the field.

We have applied MERLIN to many datasets to demonstrate its utility in generating novel biological hypotheses. The investigation into SCZ and IDPs provided causal evidence for specific bidirectional relationships. Importantly, MERLIN highlighted significant sex differences in the impact of SCZ on brain structures, such as the paracentral lobule and nucleus accumbens, suggesting that sex-specific neuropathological pathways underly clinical differences^23,34-36^. MERLIN’s analysis of testosterone effects also revealed a compelling male-specific causal risk for BD that aligned with clinical prevalence patterns^42,43^ and identified hormonal pathways that warrant further investigation^42,44,45^. Furthermore, the analysis of metabolic factors and CAD demonstrated MERLIN’s ability to quantify age-dependent effects, revealing that the causal effect of BMI varies significantly with age, underscoring the potential for age-tailored prevention.

Our study has some limitations. First, like all MR methods, MERLIN’s validity relies on core MR assumptions^4^ (i.e., IV relevance, exclusion restriction, InSIDE) being met that are further extended to the interaction context, and violations of these assumptions can still lead to bias. Second, the current MERLIN framework primarily models each interaction effect using a specific functional form (typically linear multiplicative, captured by the X×E term) and may not fully capture more complex or non-linear interaction patterns between the exposure and modifier. Third, MERLIN assumes that the modifier E is not correlated with the instrumental variables (G) used for the exposure X, restricting its application in scenarios where E itself has a strong genetic component that potentially correlates with the instruments. Future work could extend the MERLIN framework to model more flexible or non-linear interaction forms between exposures and modifiers, and crucially, to appropriately handle scenarios where the modifier (E) is genetically influenced (potentially adapting principles from multivariable MR^55^ or causal mediation analysis^56^). Finally, it remains important to benchmark the generalizability of MERLIN in diverse ancestral populations and a wider range of exposures, modifiers, and outcomes.

In conclusion, MERLIN addresses a critical need in causal inference by providing a robust, statistically principled, and broadly applicable framework for estimating both the average and heterogenous causal effects using only summary-level data. This method enhances our ability to dissect the context-dependent mechanisms underlying complex traits and diseases, paving the way for more nuanced biological insights and data-driven precision medicine.

## Methods

### MERLIN framework

MERLIN jointly estimates the average ($\beta^{\left(A\right)}$) and interaction ($\beta^{\left(I\right)}$) causal effects using GWAS and GWIS summary statistics. It models effect modification by a mean-centered variable E, which is assumed to be independent of the exposure’s instruments (G). The framework is built upon a linear structural equation model that considers M genetic variants as potential instruments (details on this model and its more generalized forms are presented in **Supplementary Notes**). For an individual i:

(1) $$X_{i}=\sum_{j=1}^{M}G_{ij}\gamma_{j}^{\left(G\right)}+E_{i}\gamma^{\left(E\right)}+\sum_{j=1}^{M}G_{ij}E_{i}\gamma_{j}^{\left(GI\right)}+\sum_{j=1}^{M}U_{ij}\eta_{X}+\epsilon_{X_{i}},$$

(2) $$Y_{i}=X_{i}\beta^{\left(A\right)}+\sum_{j=1}^{M}G_{ij}\beta_{j}^{\left(G\right)}+E_{i}\beta^{\left(E\right)}+X_{i}E_{i}\beta^{\left(I\right)}+\sum_{j=1}^{M}U_{ij}\eta_{Y}+\epsilon_{Y_{i}},$$

where $G_{ij}$, denotes the centered genotype of the j-th SNP, $E_{i}$ is the centered modifier variable, $X_{i}$ and $Y_{i}$ are the covariate-adjusted exposure and outcome traits, $U_{i}$ is the unmeasured confounder and is assumed to be independent of $G_{ij}$ and $E_{i}$, and $\epsilon_{X}$ and $\epsilon_{Y}$ are random error terms that are independent of all other terms. In this framework, $\gamma_{j}^{\left(G\right)}$ is the average effect of SNP j on exposure $X,\gamma^{\left(E\right)}$ is the average effect of environment E on exposure X, $\gamma_{j}^{\left(GI\right)}$ is the SNP-by-E interaction effect of SNP j on exposure X, $\beta^{\left(A\right)}$ is the average causal effect of X on Y, $\beta_{j}^{\left(G\right)}$ is the horizontal pleiotropic effect of SNP j on outcome Y, $\beta^{\left(E\right)}$ is the effect of modifier E on outcome Y, $\beta^{\left(I\right)}$ is the causal interaction which quantifies how the effect of X on Y is modified by E, and $\eta_{X}$ and $\eta_{Y}$ are the effects of confounder U on X and Y, respectively. The main parameters of interest in our study are $\beta^{\left(A\right)}$ and $\beta^{\left(I\right)}$.

### Summary statistics input

The causal effect of X on Y for an individual i under a given modifier level $E_{i}$ is $\beta^{\left(A\right)}+E_{i}\beta^{\left(I\right)}$. The primary goal of MERLIN is to estimate $\beta^{\left(A\right)}$ and $\beta^{\left(I\right)}$ using four sets of input summary statistics: exposure GWAS, i.e., ${\hat{\gamma }}_{j}^{\left(G\right)}$, the estimated additive effect of SNP j on exposure X, and its standard error $se\left({\hat{\gamma }}_{j}^{\left(G\right)}\right)$; exposure GWIS: ${\hat{\gamma }}_{j}^{\left(GI\right)}$, the estimated SNP-by-E interaction effect of SNP j and environment E on exposure X, and its standard error $se\left({\hat{\gamma }}_{j}^{\left(GI\right)}\right)$; outcome GWAS: ${\hat{\Gamma }}_{j}^{\left(G\right)}$, the estimated additive effect of SNP j on outcome Y, and its standard error $se\left({\hat{\Gamma }}_{j}^{\left(G\right)}\right)$; and outcome GWIS: ${\hat{\Gamma }}_{j}^{\left(GI\right)}$, the estimated $G_{j}E$ interaction effect of SNP j and environment E on exposure X, and its standard error $se\left({\hat{\Gamma }}_{j}^{\left(GI\right)}\right)$. We also provide a strategy to estimate $\beta^{\left(A\right)}$ and $\beta^{\left(I\right)}$ without the outcome GWIS, which can be of practical convenience when such summary statistics data are unavailable. We show details of this in the **Supplementary Notes**.

### Likelihood function of summary statistics

The joint likelihood function for the observed summary statistics across all IVs forms the basis of MERLIN (details in **Supplementary Notes**). We can approximate the distribution of the exposure GWAS and GWIS effect estimates (denoted as vectors ${\hat{\gamma }}^{\left(G\right)}$ and ${\hat{\gamma }}^{\left(GI\right)}$) with

(3) $${\hat{\gamma }}^{\left(G\right)}\;|\;\gamma \overset{A}{∼}N\left(S_{1}RS_{1}^{-1}\gamma^{\left(G\right)},S_{1}RS_{1}\right),$$

(4) $${\hat{\gamma }}^{\left(GI\right)}\;|\;\gamma \overset{A}{∼}N\left(S_{2}RS_{2}^{-1}\gamma^{\left(\mathrm{GI}\right)},S_{2}RS_{2}\right),$$

where $\gamma^{\left(G\right)}$ and $\gamma^{\left(\mathrm{GI}\right)}$ are vectors of true underlying genetic effects, $S_{1}$ and $S_{2}$ are diagonal matrices of the standard errors of ${\hat{\gamma }}^{\left(G\right)}$ and ${\hat{\gamma }}^{\left(GI\right)}$ respectively, and R is the linkage disequilibrium (LD) matrix among IVs.

The outcome summary statistics, i.e., GWAS statistics ${\hat{\Gamma }}^{\left(G\right)}$ and GWIS statistics ${\hat{\Gamma }}^{\left(\mathrm{GI}\right)}$, are estimates of the corresponding true underlying genetic effects on Y ($\Gamma^{\left(G\right)}$ and $\Gamma^{\left(\mathrm{GI}\right)}$). Based on our structure equation model (Equation 1 and 2), it can be shown that these effect parameters can be denoted as $\Gamma^{\left(G\right)}=\beta^{\left(A\right)}\gamma^{\left(G\right)}+\beta^{\left(G\right)}$ and $\Gamma^{\left(GI\right)}=\beta^{\left(A\right)}\gamma^{\mathrm{GI}}+\beta^{\left(I\right)}\gamma^{\left(G\right)}$. If the outcomes GWAS/GWIS do not have any sample overlap with the exposures GWAS/GWIS (derivations under sample overlap are shown in **Supplementary Notes**), the approximate distributions used to formulate the likelihood can be determined based on the nature of the modifier E.

1) For a discrete binary modifier E (values $\sqrt{\frac{1-p}{p}}$, $\sqrt{-\frac{p}{1-p}}$), which is a standardized Bernoulli random variable with probability p of being 1 and 1−p of being 0:

(5) $${\hat{\Gamma }}^{\left(G\right)}\;|\;\gamma \overset{A}{∼}𝒩\left(S_{3}RS_{3}^{-1}\left(\Gamma^{\left(G\right)}+\beta^{\left(I\right)}\gamma^{\left(\mathrm{GI}\right)}\right),S_{3}RS_{3}\right),$$

(6) $${\hat{\Gamma }}^{\left(\mathrm{GI}\right)}\;|\;\gamma \overset{A}{∼}𝒩\left(S_{4}RS_{4}^{-1}\Gamma^{\left(\mathrm{GI}\right)}+\mu_{3}\beta^{\left(I\right)}S_{4}^{2}S_{3}^{-1}RS_{3}^{-1}\gamma^{\left(\mathrm{GI}\right)},S_{4}RS_{4}\right),$$

where $S_{3}$ and $S_{4}$ are diagonal matrices of the standard errors, and $\mu_{3}=\frac{1-2p}{\sqrt{p(1-p)}}$.

2) For a continuous modifier E with zero skewness $\left(\mu_{3}=0\right)$:

(7) $${\hat{\Gamma }}^{\left(G\right)}\;|\;\gamma \overset{A}{∼}𝒩\left(S_{3}RS_{3}^{-1}\Gamma^{\left(G\right)}+S_{3}^{2}S_{4}^{-1}RS_{4}^{-1}\beta^{\left(I\right)}\gamma^{\left(\mathrm{GI}\right)},S_{3}RS_{3}\right),$$

(8) $${\hat{\Gamma }}^{\left(\mathrm{GI}\right)}\;|\;\gamma \overset{A}{∼}𝒩\left(S_{4}RS_{4}^{-1}\Gamma^{\left(\mathrm{GI}\right)},S_{4}RS_{4}\right).$$

Although these forms are derived under the assumption of zero skewness, we demonstrate in the **Supplementary Notes** that hypothesis testing for the interaction effect remains robust to violations of this assumption.

### Bayesian hierarchical model and inference

MERLIN employs a Bayesian hierarchical model. The true per-SNP genetic effects are assigned prior distributions:

$$\gamma^{\left(G\right)}∼N(0,\sigma_{1}^{2}I),\;\beta^{\left(G\right)}∼N(0,\sigma_{2}^{2}I),\;\gamma^{\left(\mathrm{GI}\right)}∼N(0,\sigma_{3}^{2}I).$$

The variance component $\sigma_{k}^{2}$ is assigned appropriate hyperpriors: inverse-gamma distributions $\mathrm{InvGamma}(a_{k},b_{k})$, k=1,2,3. Non-informative prior distributions are placed on the primary parameters $\beta^{\left(A\right)}$ and $\beta^{\left(I\right)}$. Posterior distributions for all unknown parameters are obtained using Markov Chain Monte Carlo (MCMC) methods. Full details of the MCMC algorithm implemented in MERLIN are provided in **Supplementary Notes**.

### IV selection strategy

The IV selection strategy employed by MERLIN involves selecting IVs if they meet the significance threshold in either exposure GWAS ($P<T_{\mathrm{GWAS}}$) or exposure GWIS ($P<T_{\mathrm{GWIS}}$), followed by LD clumping (e.g., $r^{2}<0.01$, 1Mb window). For any SNP that is significant in both GWAS and GWIS, the minimum P value is used to prioritize it during the clumping process. The specific thresholds $T_{\mathrm{GWAS}}$, $T_{\mathrm{GWIS}}$ and $r^{2}$ values are adapted for each application to reflect the signal strengths in the input data, as we detail in the next section.

### Simulation setup

Simulations were based on real genotypes from 337,056 unrelated UKB individuals of European ancestry (102,112 common SNPs, MAF > 0.01, chromosome 1). For each simulation replicate, we constructed two separate, non-overlapping cohorts: an exposure cohort of 80,000 individuals and an outcome cohort of 80,000 individuals. To ensure a balanced distribution for sex as a modifier, each of these cohorts was composed of 40,000 males and 40,000 females. Within each cohort, 200 independent SNPs were randomly chosen to act as potential causal IVs. Exposure (X) and outcome (Y) trait values were then simulated for these respective cohorts based on the MERLIN linear structural model (see Equations 1 and 2). Summary statistics were subsequently obtained through GWAS and GWIS analyses for the simulated traits. For the exposure cohort, GWAS and GWIS summary statistics were generated from the same 80,000 individuals, representing a complete sample overlap between two exposure summary statistics. The same procedure was applied to the analysis of the outcome cohort. Note that the MERLIN framework was theoretically designed to be robust to any degree of overlap between GWAS and GWIS for a given trait (i.e., no, partial, or full overlap). This setup, where exposure and outcome cohorts are independent, but GWAS/GWIS data for a given trait are from the same individuals, mimics many real-world application scenarios and is one of many conditions that MERLIN can accommodate.

Each simulation scenario was repeated 500 times. We fixed the proportion of variance in X explained by the average genetic effects ($h_{\gamma^{\left(G\right)}}^{2}$) at 0.3, and varied several key parameters across different simulation scenarios: GWIS signal strength for exposure $\left(h_{\gamma^{\left(\mathrm{GI}\right)}}^{2})=(0.1,0.15,0.3\right)$, corresponding to (1/3,1/2,1) times $h_{\gamma^{\left(G\right)}}^{2}$, respectively; correlation between GWAS ($\gamma^{\left(G\right)}$) and GWIS ($\gamma^{\left(\mathrm{GI}\right)}$) ($\rho_{A-I}=0,0.4,0.8$); and horizontal pleiotropy level ($h_{\beta^{\left(G\right)}}^{2}=0,0.05,0.1$).

The non-genetic components contributing to the variance of X and Y were defined as $e_{X_{i}}=E_{i}\gamma^{\left(E\right)}+U_{i}\eta_{X}+\epsilon_{X_{i}}$ and $e_{Y_{i}}=E_{i}\beta^{\left(E\right)}+U_{i}\eta_{Y}+\epsilon_{Y_{i}}$. The variances of these residual terms were scaled such that the total variances of the simulated phenotypes X and Y were both 1. The correlation between these residual terms was set to 0.6 to mimic the phenotypic correlation potentially induced by the shared modifier E and confounder U. Both discrete (binary, coded ±1) and continuous (standard normal) modifiers E were simulated.

Additionally, to test the robustness of MERLIN to unbalanced discrete modifiers, a separate set of simulations was conducted under a simplified setting ($\rho_{A-I}=0$, $h_{\beta^{\left(G\right)}}^{2}=0$). In this scenario, the binary modifier E was generated from an unbalanced distribution of 25% males and 75% females.

To evaluate performance in the presence of exposure–outcome sample overlap, we conducted simulations where the overlap between the exposure and outcome cohorts was fixed at 40,000 individuals, composed of 20,000 males and 20,000 females. This sample overlap condition was applied across various simulation frameworks, all of which were run in the absence of horizontal pleiotropy ($h_{\beta^{\left(G\right)}}^{2}=0$). These frameworks included null models ($\beta^{\left(A\right)}=0$ and $\beta^{\left(I\right)}=0$) to assess type I error control, as well as scenarios with non-zero effects ($\beta^{\left(A\right)}=0.3$ or $\beta^{\left(I\right)}=0.3$) to evaluate estimation accuracy and statistical power.

For each simulated setting, GWAS and GWIS summary statistics were generated using PLINK. These generated summary statistics served as input for MERLIN, the MR methods (MR-LDP, RAPS, IVW, MR-Egger), and sex-stratified IVW (for scenarios with discrete E). The estimation bias, type I error rate, and statistical power of these methods were compared.

### Data sources and genomic data processing

We analyzed genomic data from UKB (https://www.ukbiobank.ac.uk) and the All of Us Research Program (https://researchallofus.org). We also used summary statistics data from the Psychiatric Genomics Consortium (PGC) (https://pgc.unc.edu/for-researchers/download-results), Genetic Investigation of ANthropometric Traits (GIANT) consortium (https://giant-consortium.web.broadinstitute.org/GIANT_consortium), Oxford Brain Imaging Genetics Server (https://open.win.ox.ac.uk/ukbiobank/big40), and Winkler et al. (2024)^47^ in various applications.

Although the MERLIN framework is theoretically equipped to handle unbalanced binary modifiers (see **Supplementary Notes** for the general likelihood derivation), a valid two-sample design requires the modifier’s distribution to be consistent across the exposure and outcome cohorts. Therefore, in our primary analyses, we aligned our study with our main data source, the UKB, by selecting traits for sex-interaction analysis only if the proportion of males (p) in their effective sample size was between 0.4 and 0.6 (i.e., ∣p−0.5∣<0.1).

Additional quality control involved removing SNPs with MAF < 0.05. Following Bulik–Sullivan, et al.^57^, we further filtered GWAS and GWIS data by selecting SNPs with $Z^{2}<\max(80,N∕1000)$. For each exposure–outcome pair analyzed, overlapping SNPs present in both summary statistics were extracted. Effect estimates for these SNPs were subsequently aligned to ensure harmonization based on the same reference allele across datasets. **Supplementary Table S1-2** details dataset sources and sample sizes.

### Generation of GWIS and sex-combined GWAS from stratified data

For analyses requiring G×Sex GWIS where only sex-stratified GWAS were available (e.g., for some PGC datasets or UKB testosterone for certain applications), GWIS summary statistics were derived following^19^

$$\;{\hat{b}}_{\text{gwis}\;j}\;=\frac{1}{2}({\hat{b}}_{M,j}-{\hat{b}}_{F,j}),\;\text{s e}({\hat{b}}_{\text{gwis}\;j})\;=\frac{1}{2}\sqrt{\text{s e}{\left({\hat{b}}_{M,j}\right)}^{2}+\text{s e}{\left({\hat{b}}_{F,j}\right)}^{2}},$$

where ${\hat{b}}_{M,j}$, $se\left({\hat{b}}_{M,j}\right)$ and ${\hat{b}}_{F,j}$, $se\left({\hat{b}}_{F,j}\right)$ are the SNP effects and standard errors from male-specific and female-specific GWAS, respectively.

For traits with sex-stratified GWAS but lacking sex-combined GWAS, the latter were generated by meta-analyzing male and female summary statistics using the inverse-variance weighted approach implemented in METAL^58^.

### GWAS and G×Age GWIS for CAD in All of Us

CAD GWAS and SNP-by-age interaction (G×Age) GWIS summary statistics were generated using data from European ancestry participants in the All of Us Research Program^59^ v8 data release. CAD status was defined using a standard set of ICD-10 codes, including angina (I20), myocardial infarction (MI, I21, I22), complications following MI (I23), status post-acute MI (I253), coronary atherosclerosis (I24, I25, Z951, T822), and coronary revascularization, following an established methodology^60^. Standard GWAS analyses using PLINK2^61^ were adjusted for sex, age, and the top 16 principal components (PCs). Corresponding G×Age GWIS was used to estimate the SNP×age interaction effects, adjusting for sex and the top 16 PCs.

### Additional data pre-processing for the negative control analysis

For the BMI and WHR negative control analyses, the original sex-stratified UKB GWAS summary statistics were scaled by dividing the estimates by the standard deviation of UKB samples^62^. This ensured there was consistency in the phenotype definition. GIANT consortium data were used as the outcome.

### IV selection in real data applications

For the majority of analyses, IVs were selected based on exposure-specific summary statistics and using the 1000 Genomes Project Phase 3 European panel as the LD reference. However, for specific analyses of metabolic biomarkers on CAD, we utilized the HapMap 3 reference panel. This modification was made to maximize the number of available instruments and ensure compatibility with the downstream CAD summary statistics. Distinct selection parameters were applied for the comparator methods versus the MERLIN framework.

For MR methods that assume IV independence (IVW, MR-Egger, RAPS), IVs were selected from the exposure GWAS at a genome-wide significance threshold ($P<5\times 10^{-8}$) and pruned for LD using a strict clumping threshold ($r^{2}<0.01$, 1 Mb window). For MR-LDP, which is designed to accommodate correlated IVs, a relaxed LD threshold of $r^{2}<0.3$ was generally used to enhance statistical power.

For MERLIN, which leverages instruments from both GWAS and GWIS, we applied a consistent significance threshold for GWAS instruments ($T_{\mathrm{GWAS}}=5\times 10^{-8}$) and a general LD clumping threshold of ($r^{2}<0.3$). Recognizing that G×E interaction signals from GWIS are often weaker than the average effects from GWAS, the significance threshold for GWIS instruments ($T_{\mathrm{GWIS}}$) was adapted based on the expected signal strength for each exposure. Specifically, a more stringent threshold ($T_{\mathrm{GWIS}}=5\times 10^{-8}$) was used for analyses with stronger expected interaction signals (analyses of testosterone–neuropsychiatric disorders), while a moderately relaxed threshold ($T_{\mathrm{GWIS}}=5\times 10^{-6}$) was used for the other analyses.

### MR methods applied in real data applications

The primary analytical tool used to jointly estimate the average effect ($\beta^{\left(A\right)}$) and the interaction effect ($\beta^{\left(I\right)}$) was the MERLIN framework. For analysis involving sex as a modifier, sex-specific effects were derived from MERLIN estimates using ${\hat{\beta }}_{M}={\hat{\beta }}^{\left(A\right)}+{\hat{\beta }}^{\left(I\right)}$ and ${\hat{\beta }}_{F}={\hat{\beta }}^{\left(A\right)}-{\hat{\beta }}^{\left(I\right)}$ (assuming sex coded as Male = 1, Female = –1; see **Supplementary Notes** for details).

For comparison purposes, standard MR methods (IVW, MR-Egger, RAPS, and MR-LDP) were also applied to estimate the average effect, implemented using the software detailed below. Additionally, sex-stratified IVW, RAPS, MR-Egger, MR-LDP were performed as a comparator for the interaction effect. This involved applying those methods separately to male- and female-specific GWAS summary statistics (using instruments selected specifically within each stratum, as detailed in IV selection in the data analysis section) to obtain stratified causal estimates for males ($\beta_{M}$) and females ($\beta_{F}$). The interaction effect parameter, corresponding conceptually to the $\beta^{\left(I\right)}$ estimated by MERLIN, was then derived from these stratified estimates using the formula: ${\hat{\beta }}^{\left(I\right)}=0.5({\hat{\beta }}_{M}-{\hat{\beta }}_{F})$.

### Sensitivity analyses for real data applications

Sensitivity analyses were performed for MERLIN under different IV selection thresholds.

(1) Negative and Positive Control: Re-analyzed under 27 combinations of IV selection thresholds (3 $r^{2}$ thresholds [0.01, 0.1, 0.3]; 3 $T_{\mathrm{GWAS}}$ values [5 × 10^−8^, 1 × 10^−7^, 5 × 10^−7^]; 3 $T_{\mathrm{GWIS}}$ values [5 × 10^−6^, 1 × 10^−5^, 5 × 10^−5^]).

(2) SCZ-IDPs: Two significant interaction pairs evaluated under 27 settings (3 $r^{2}$ thresholds [0.1, 0.3, 0.5]; 3 $T_{\mathrm{GWAS}}$ values [5 × 10^−8^, 1 × 10^−7^, 5 × 10^−7^]; 3 $T_{\mathrm{GWIS}}$ values [5 × 10^−6^, 1 × 10^−5^, 5 × 10^−5^]).

(3) Testosterone-BD: Sex interaction evaluated under under 27 settings (3 $r^{2}$ thresholds [0.1, 0.3, 0.5]; 3 $T_{\mathrm{GWAS}}$ and $T_{\mathrm{GWIS}}$ values [5 × 10^−8^, 1 × 10^−7^, 5 × 10^−7^]).

(4) BMI-CAD: Age interaction evaluated under 27 settings (3 $r^{2}$ thresholds [0.1, 0.3, 0.5]; 3 $T_{\mathrm{GWAS}}$ values [5 × 10^−8^, 1 × 10^−7^, 5 × 10^−7^]; 3 $T_{\mathrm{GWIS}}$ values [5 × 10^−6^, 1 × 10^−5^, 5 × 10^−5^]).

(5) Metabolic biomarkers-CAD: Sensitivity analyses were performed for the age interactions under 27 settings (3 $r^{2}$ thresholds [0.1, 0.3, 0.5] ; 3 $T_{\mathrm{GWAS}}$ values [5 × 10^−8^, 1 × 10^−7^, 5 × 10^−7^]; 3 $T_{\mathrm{GWIS}}$ values [5 × 10^−6^, 1 × 10^−5^, 5 × 10^−5^]).

## Supplementary Material

This is a list of supplementary files associated with this preprint. Click to download.

- 3SupplementaryTables.xlsx
- 2SupplementaryNotesandFigures.pdf

## Data Availability

All GWAS summary statistics used in this study are publicly available. Specific dataset sources and URLs are provided in **Supplementary Table S1**. The 1000 Genomes Project Phase 3 reference panel is publicly available at https://www.ebi.ac.uk/ega/. Data supporting the tutorial of the developed R package can be downloaded from Figshare (https://figshare.com/articles/dataset/Data_for_MERLIN/29910116).

## Code availability

The “MERLIN” R package is freely available at https://github.com/shilab-ecnu/MERLIN. LD clumping was performed using PLINK (v.2.0)^61^. Meta-analyses used METAL (v.2011-03-25)^58^. Genetic correlations used LDSC (v.1.0.1)^57^. Standard MR analyses were implemented using the following R packages: “TwoSampleMR” (v.0.6.6)^63^ for IVW and MR-Egger, “mr.raps” (v.0.4.1)^6^ for RAPS, and “MR.LDP” (v.1.0)^5^ for MR-LDP. Analysis pipelines and scripts for replicating all results presented in this paper are available at https://github.com/shilab-ecnu/MERLIN.
